# Molecular Dynamic Simulation Reveals Damaging Impact of RAC1 F28L Mutation in the Switch I Region

**DOI:** 10.1371/journal.pone.0077453

**Published:** 2013-10-16

**Authors:** Ambuj Kumar, Vidya Rajendran, Rao Sethumadhavan, Rituraj Purohit

**Affiliations:** 1 Bioinformatics Division, School of Bio Sciences and Technology, Vellore Institute of Technology University, Vellore, Tamil Nadu, India; 2 Human Genetics Foundation, Torino, Torino, Italy; Institut Jacques Monod, France

## Abstract

Ras-related C3 botulinum toxin substrate 1 (RAC1) is a plasma membrane-associated small GTPase which cycles between the active GTP-bound and inactive GDP-bound states. There is wide range of evidences indicating its active participation in inducing cancer-associated phenotypes. RAC1 F28L mutation (RAC^F28L^) is a fast recycling mutation which has been implicated in several cancer associated cases. In this work we have performed molecular docking and molecular dynamics simulation (~0.3 μs) to investigate the conformational changes occurring in the mutant protein. The RMSD, RMSF and NHbonds results strongly suggested that the loss of native conformation in the Switch I region in RAC1 mutant protein could be the reason behind its oncogenic transformation. The overall results suggested that the mutant protein attained compact conformation as compared to the native. The major impact of mutation was observed in the Switch I region which might be the crucial reason behind the loss of interaction between the guanine ring and F28 residue.

## Introduction

**Figure 1:**
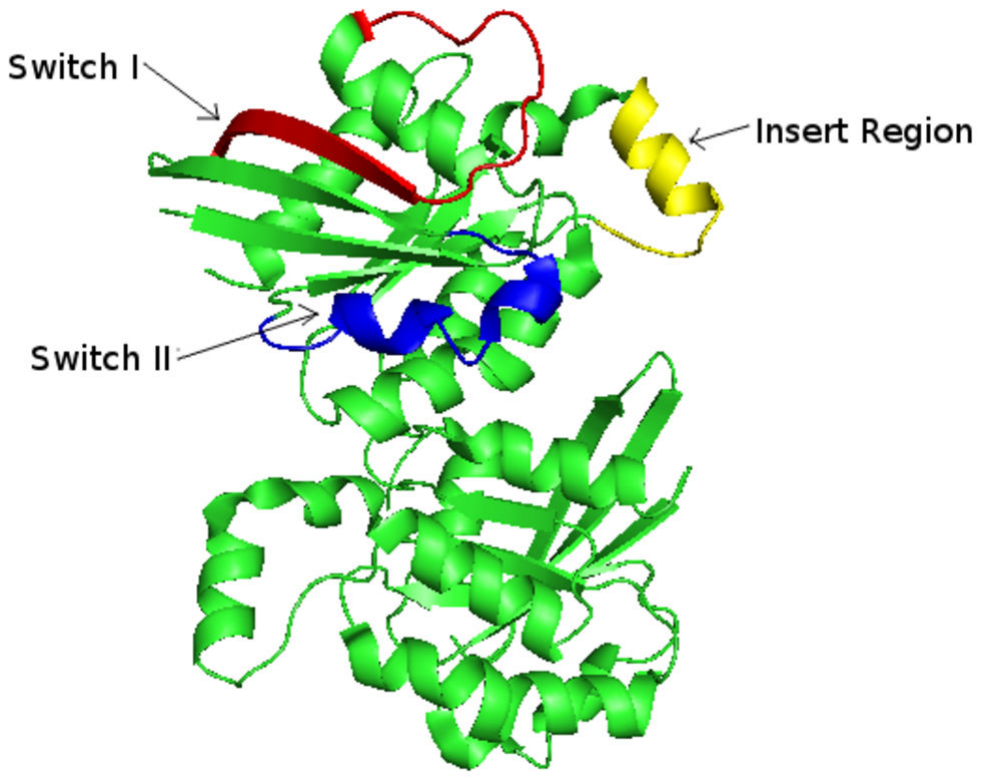
Representation of Switch I, Switch II and Insert region in RAC1 protein.

 RAC1 is a small, Ras-related GTPase belonging to the Rho family functions as a binary molecular switch, cycling between an inactive GDP-bound “OFF” state and an active GTP-bound “ON” state [1]. Its assists in the regulation of various cellular activities including NADPH oxidase activation, secretory processes, phagocytosis of apoptotic cells, epithelial cell polarization, formation of cortical actin-containing membrane ruffles and lamellipodia, and induction of gene expression programs [1,2]. It is essential for the SPATA13-mediated regulation of cell migration and adhesion assembly and disassembly. It's other biological processes involve cell proliferation, cell-cell junction organization, cell-matrix adhesion, dendrite morphogenesis, negative regulation of interleukin-23 production, negative regulation of receptor-mediated endocytosis and positive regulation of lamellipodium assembly [3-6]. RAC1 protein consists of three functional regions that include Switch I, Switch II and the Insert region (Figure 1). The Switch regions in RAC1 are the most important structural element of the protein. The Switch regions consist of Switch I and Switch II. Switch I contains residues 26-45 and Switch II contains residues 59-74. These regions are responsible for the molecular interactions of RAC1, except those that deal with membrane interactions. Switch I primarily interacts with downstream effectors, such as IQGAP1 and proteins in the NADPH complex. Because of its close affinity with downstream effectors, Switch I region is hence known as the "effector region". Switch II, on the other hand, interacts with RAC1 activating proteins, or GEFs. The Switch II region is the site where RAC1 becomes activated in its GTP-bound state. The Insert Region of RAC1 consists of residues 124-135. This region is only present in the Rho subfamily of GTPases, and therefore is a distinct element of Rho GTPases. The Insert Region is located between beta-strand 5 and alpha-helix 4. This region is essential for mitogenesis and apoptosis. It also plays a significant role in regulating interactions with downstream effectors, specifically in the NADPH complex. The c-terminus participates in the binding of RAC1 to the membrane. It is particularly important in the NADPH complex, where RAC1 binds to the membrane to facilitate the production of superoxide. Members of the Rho subfamily of GTPases share approximately 92% sequence homology. The divergence, however, occurs mainly in the c-termini. The c-terminus in RAC1 contains polybasic amino acid residues, whereas other Rho family proteins are less basic.

 Series of phosphorylation and autophosphorylations are mediated by various protein interactions in RAC1. A number of RAC1 residues, Thr35Rac, Tyr64Rac, Arg66Rac, His103Rac, and His104Rac, are involved in hydrogen bonds with RhoGDI [7]. The interaction between Thr35Rac and Asp45GDI is particularly important for inhibition of the GDP-GTP exchange [7]. In the RAC1-RhoGDI structure, Rac1 is in a GDP conformation with the carbonyl of Thr35^Rac^ coordinating to Mg^2+^ [7]. In the interaction with RhoGDI, GDP is strongly stabilized by a hydrogen bond between the hydroxyl group of Thr35^Rac^ [7]. Other residues such as Tyr64^Rac^, Arg66^Rac^, His103^Rac^, His104^Rac^, Leu67^Rac^, Leu70^Rac^, located in the switch II region of RAC1, help in the interactions with RhoGDI maintain this region in a GTP-like conformation despite the fact that RAC1 binds a GDP molecule [7]. Rac undergoes a posttranslational modification that allows its localization to the plasma membrane [8]. It consists of the covalent binding of a geranylgeranyl group to the cysteine residue of c-terminal CAAX motif via a thioether linkage, a reaction that is catalyzed by a geranylgeranyl transferase [8]. 

 RAC1 activity has been reported in regulating various pathways of oncogenesis including initiation, progression, invasion, and metastasis [1]. Overexpression of RAC1 has been reported in multiple cases of colorectal, pancreatic, breast, testicular cancers and in several leukemia cases [1]. Moreover, a self-activating splice variant of RAC1, RAC1b, was shown to be overexpressed in breast cancer and lung cancer and is thought to mediate the epithelial–mesenchymal transition in lung epithelial cells [1]. Furthermore, aberrant activation of upstream regulators of RAC1, particularly in the DBL family of GEFs specific for RAC1, has been implicated in various cancers [1]. It has been recently reported that targeting of RAC1 protein suppresses human non-small cell lung adenocarcinoma cancer stem cell activity [9]. Authors showed that the RAC1 knockdown prevented lung colonization of NSCLA cells in mice [9]. Moreover, the subcutaneous xenograft of the tumor cells in NSG mice showed that the RAC1 knockdown cells had delayed tumor development and reduced tumor volume compared with the control cells [9]. By the collective observations, authors proposed that there are likely chances of RAC1 knockdown to affect the tumor cell lung colonization, growth due to a combined effect on cancer cell homing and proliferation in the lung [9]. In other studies, the immunohistochemical staining of RAC1 showed weak RAC1 expression in benign breast disease but high expression level in ductal carcinoma-in-situ, primary breast cancer, and lymph node metastases [10]. In addition, breast tumor cells from patients with recurrent disease had RAC1 expression at the plasma membrane, suggesting activation of RAC1, in patients with aggressive breast cancer [10]. Moreover, in a recent investigation, a recurrent somatic missense mutation at codon 29 of RAC1 that results in substitution of a proline to a serine residue (RAC1^P29S^) was discovered that up to 9% of sun-exposed melanomas and is considered as the most common cancer-associated recurrent missense mutation in a Rho family GTPase. Another RAC1 mutation F28L has also been observed in several cancer cases [1]. The F28L mutation in RAC1 also results in loss of interaction between codon 28 and the nucleoside, suggesting that for RAC1^F28L^, fast cycling results from reduced affinity for nucleotide [1]. Structural and biochemical data suggest that RAC1^F28L^ and RAC1^P29S^ are self-activated by different mechanisms, with RAC1^F28L^ self-activation driven by a loss in interaction between the guanine ring and F28, and RAC1^P29S^ is possibly driven by another mechanism, perhaps destabilization of the GDP-loaded inactive state [1]. Although the overall architectures are very similar, the conformation of the Switch I loops of RAC1^P29S^ and RAC1^F28L^ are divergent from each other, with RAC1^P29S^ showing a Ras-like Switch I conformation and RAC1^F28L^ displaying increased flexibility [1]. For RAC1^F28L^, this is probably due to the loss of the phenylalanine benzyl group and consequent reduced stabilizing interactions with nucleotide [1].

 Investigating the structural consequences induced by disease-associated genetic mutations will provide a significant knowledge of associated conformational changes occurring in the functionally significant regions of the protein. Since RAC1F28L mutation has been widely indicated in several cases of cancers and has been reported to cause alterations in the ligand binding affinity and flexibility level of the Switches, the application of computational molecular docking and molecular dynamic simulation approaches can be very useful in obtaining the underlying molecular insights of the associated phenotypic outcomes. As we know that conformational changes in the protein structure affect its biological function. It is also well evident that the conformational flexibility of a protein molecule affects its interaction with ligand and its biological partners at different level [11-19]. Thus, we carried molecular docking and long term molecular dynamics simulation in order to investigate the changes in the dynamic behaviour of the protein functional region and to elucidate the molecular causes associated with the oncogenesis of mutant protein 

## Materials and Methods

### Dataset collection

 The native (PDB ID: 3TH5) [20] and F28L mutant (PDB ID: 4GZM) [1] RAC1 protein 3D structures were obtained from Protein Data Bank [21]. The native and the mutant RAC1 protein crystal structures were obtained by using X-Ray diffraction method in the work of Krauthammer et al. [20] and Davis et al. [1] works respectively. 

### Protein–ligand interaction analysis

 Molecular docking studies were performed to investigate the role of mutation over GTP-binding activity of RAC1 protein using Autodock 4.0 [22]. AutoDockTools 1.4.6 was used for establishing the Autogrid points as well as visualization of docked ligand-amino acid structures [22]. In this docking simulation, we used semi-flexible docking protocols in which the binding residues of the target protein were kept as flexible and others were kept rigid. The ligands being docked were also kept flexible, in order to explore an arbitrary number of torsional degrees of freedom in addition to the six spatial degrees of freedom spanned by the translational and rotational parameters. Grid map centred on the ligands binding sites of native and mutant structures were constructed to cover the GTP-binding pockets. Lamarckian genetic algorithm was used to carry out molecular-docking simulations. Simulations were performed using up to 2.5 million energy evaluations with a maximum of 27,000 generations. The lowest energy conformation was considered as the binding conformation between GTP and RAC1.

### Molecular dynamics simulation

Molecular dynamics simulation was performed by using Gromacs 4.5.4 package [23] running on a Linux cluster. Systems were solvated in a rectangular box with TIP3P water molecules at 10 Å marginal radius. At physiological pH, the structures were found to be negatively charged, thus in order to make the simulation system electrically neutral, we added 1 sodium ions Na^+^ in the simulation box using the ‘genion’ tool that accompanies with gromacs package and genion tool replaces solvent molecules by monoatomic ions at the position of the first atoms with the most favourable electrostatic potential or at random. The solvent molecules were first relaxed while all the solute atoms were harmonically restrained to their original positions with a force constant of 100 kcal/mol for 5000 steps. Emtol convergence criterion was set to 1000 kcal/mol. After this, whole molecular system was subjected to energy minimization by steepest descent algorithm implementing GROMOS96 43a1 force field. Berendsen temperature coupling method [24] was used to regulate the temperature inside the box. Isotropic pressure coupling was performed using Parrinello–Rahman method. Electrostatic interactions were computed using the Particle Mesh Ewald method [25]. The ionization states of the residues were set appropriate to pH 7 with all histidine assumed neutral. The pressure was maintained at 1 atm with the allowed compressibility range of 4.5e^-5^ atm. SHAKE algorithm was used to constrain bond lengths involving hydrogen, permitting a time step of 2 fs. Van der Waals and coulomb interactions were truncated at 1.0 nm. The non-bonded pair list was updated every 10 steps and conformations were stored every 0.5 ps. 

 Position restraint simulation for 20 ns was implemented to allow solvent molecules to enter the cavity region of structure. It also helps in restraining the atoms at a fixed reference position. Finally, systems were subjected to MD simulation for 300 ns. We computed the comparative analysis of structural deviations in native and mutant RAC1 structure. g_rms compares two structures by computing the root mean square deviation (RMSD), the size-independent ’rho’ similarity parameter (rho) or the scaled rho (rhosc) and the g_rmsf computes the root mean square fluctuation. g_rms, g_rmsf, g_covar and g_anaeig gromacs inbuilt tools were used for protein trajectories and atomic interaction analysis. Number of distinct hydrogen bonds formed by specific residues to other amino acids within the protein during the simulation (NHbond) was calculated using g_hbond. The program g_hbond analyses the hydrogen bonds (H-bonds) between all possible donors D and acceptors A. To determine if an H-bond exists, a geometrical criterion is used, r ≤ rHB = 0.35 nm and α ≤ αHB = 30°. The value of rHB = 0.35 nm corresponds to the first minimum of the radial distribution function of SPC water. NHbond determined on the basis of donor–acceptor distance smaller than 0.35 nm and of donor–hydrogen-acceptor. Graphs were plotted using Grace GUI toolkit 5.1.22 version.

### Principal component analysis

 The calculation of the eigenvectors and eigenvalues, and their projection along the first two principal components, was carried out using essential dynamics (ED) method according to protocol [26] within the GROMACS software package. The principle component analysis or ED is a technique that reduces the complexity of the data and extracts the concerted motion in simulations that are essentially correlated and presumably meaningful for biological function [26]. It can be used to find the correlated motions of macromolecules. In the ED analysis, a variance/covariance matrix was constructed from the trajectories after removal of the rotational and translational movements. A set of eigenvectors and eigenvalues was identified by diagonalizing the matrix. The eigenvalues represents the amplitude of the eigenvector along the multidimensional space, and the displacement of atoms along each eigenvector shows the concerted motions of protein along each direction. The movements of structures in the essential subspace were identified by projecting the Cartesian trajectory coordinates along the most important eigenvectors from the analysis. Backbone C-alpha bonds trajectories were obtained using g_covar and g_anaeig of gromacs utilities.

## Results and Discussion

 In our previous work, we have shown highly damaging structural consequences of genetic mutations on native conformations of proteins [27-32]. Here we have investigated the structural consequences of cancer associated mutation F28L in RAC1 protein. The molecular docking analysis was conducted using autodock 4.0 packages to unravel the changes in GTP-binding affinity in mutant structure as compared to native. A notable change in interaction affinity was observed in mutant structure. In native RAC1 protein, the optimal binding energy was ~ 7.32 kcal/mol whereas in mutant (F28L) it was found to be ~ 2.45 kcal/mol. The changes in interaction affinity obtained in our work in direct concordance to the result obtained by Davis et al. [1] and has revealed the damaging consequences of mutation on GTP binding affinity of RAC1 protein.

**Figure 2:**
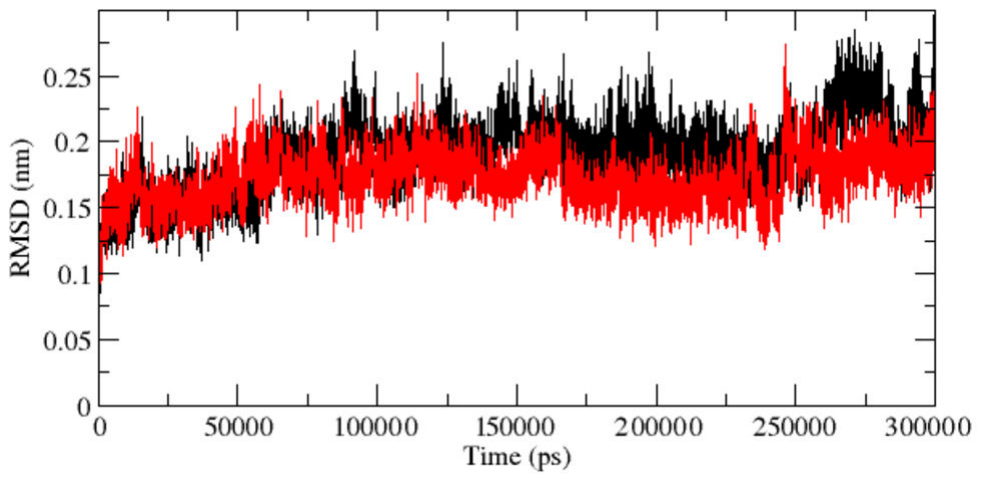
Backbone RMSDs are shown as a function of time for native and mutant RAC1 protein structures at 300 K. Native is shown in black and mutant in red.

**Figure 3:**
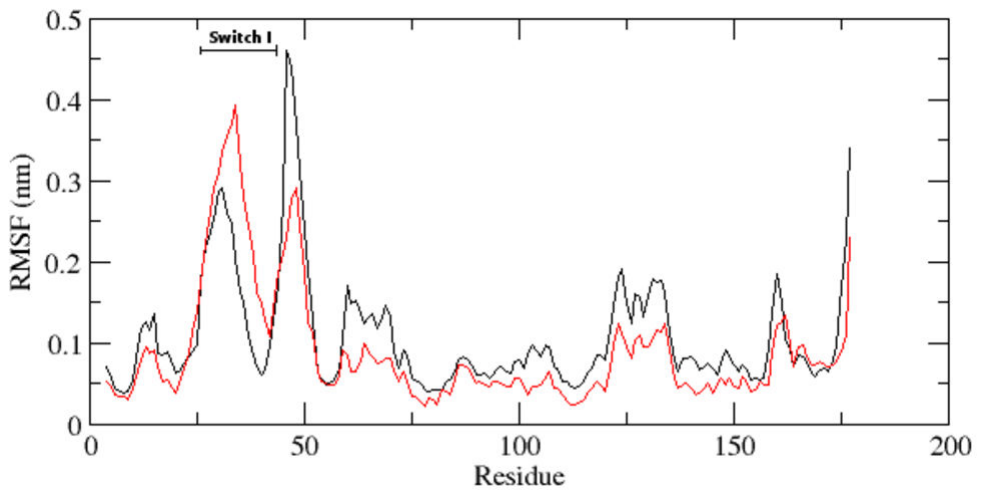
RMSF of the backbone Cα atoms of native and mutant RAC1 protein versus time at 300 K. Native is shown in black and mutant in red.

**Figure 4:**
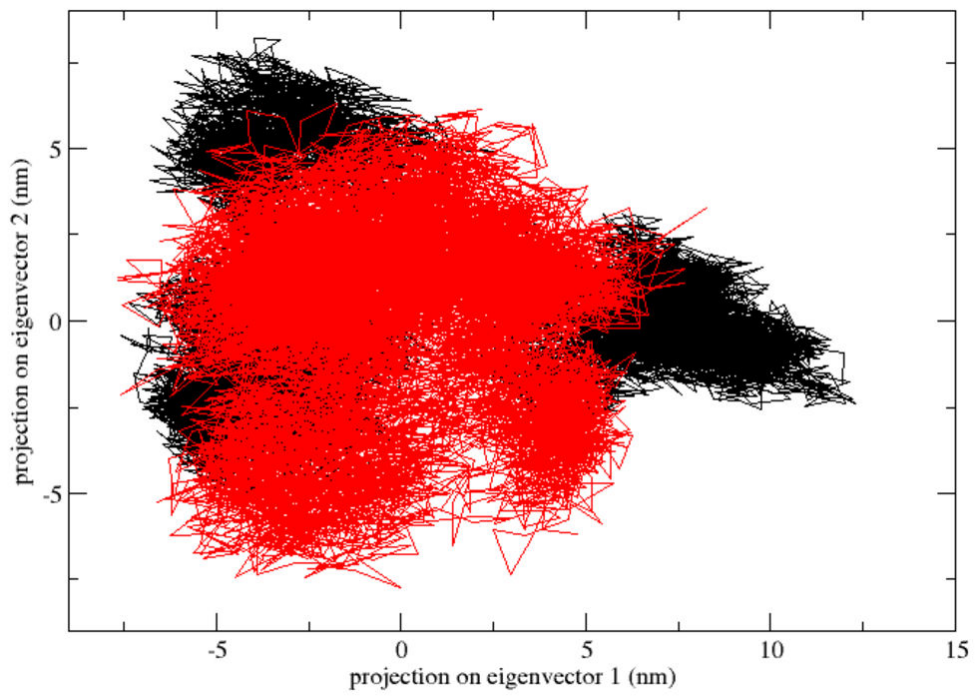
Projection of the motion of the protein in phase space along the first two principal eigenvectors for native and mutant RAC1 protein at 300 K: Native is shown in black and mutant in red.

 Protein–ligand interactions are often accompanied by significant changes in their conformation. To illustrate the molecular changes associated with the loss of ligand interaction affinity of mutant protein, it is recommended to perform large scale molecular dynamics simulations. Thus, we conducted molecular dynamics simulation for 300 ns to examine the changes in conformation behaviour of the mutant protein as compared to the native. We investigated RMSD, RMSF and NHbond variation between the native and mutant structure. RMSD for all the Cα atoms from the initial structure were calculated which was considered as the central criterion to measure the protein system. In Figure 2, native and mutant RAC1 proteins showed similar type of deviation throughout the simulation from their starting structure, resulting in backbone RMSD ~ 0.07 nm -0.29 nm during the simulation. Mutant showed slightly distinct fashion of deviation after ~170 ns when compared to native, although the overall RMSD fluctuations were very much similar in native as well as in mutant. This magnitude of fluctuations together with very small difference between the average RMSD values after the relaxation period (~0.025 nm), led to conclusion that simulation produced stable trajectories, thus providing a suitable basis for further analyses. 

**Figure 5:**
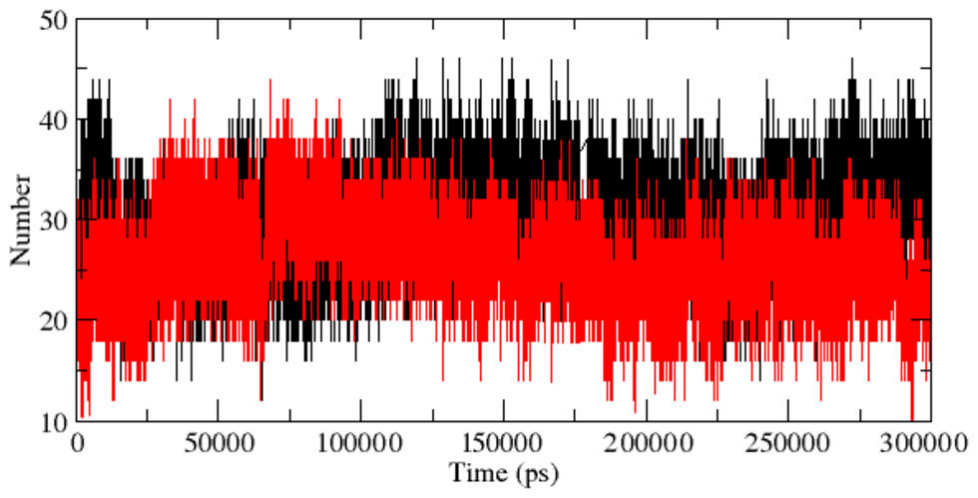
Average number of protein–solvent intermolecular hydrogen bonds in native and mutant RAC1 protein Switch I region versus time at 300 K. Native is shown in black and mutant in red.

 With the aim of determining whether mutation affected the dynamic behaviour of residues, the RMSF values of native and mutant backbone residues were calculated (Figure 3). Analysis of fluctuation score revealed the presence of higher degree of flexibility in native as compared to the mutant RAC1 protein. The presence of higher RMSF values in the native structure depicts that the F28L mutation induced constrains in the flexibility of protein structure. Moreover, it was very interesting to observe that the amino acid residues present in the Switch I region of RAC1 protein showed distinct fashion of RMSF change (Figure 3). Amino acid residues of mutant RAC1 Switch I region was shown to exhibit higher RMSF values as compared to the native. This result directly indicates that the mutation has affected the conformation of Switch I region in different fashion, which caused rise in flexibility of Switch I residues whereas it induced constrains in the flexibility of the residues in other regions. 

**Figure 6:**
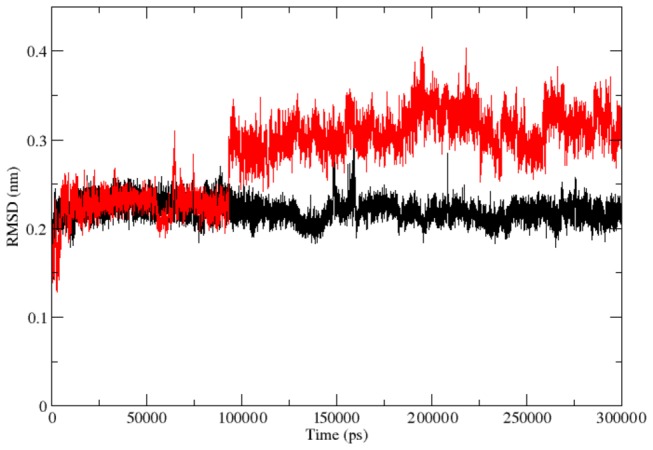
Backbone RMSDs are shown as a function of time for native and mutant RAC1 protein Switch I region at 300 K. Native is shown in black and mutant in red.

**Figure 7:**
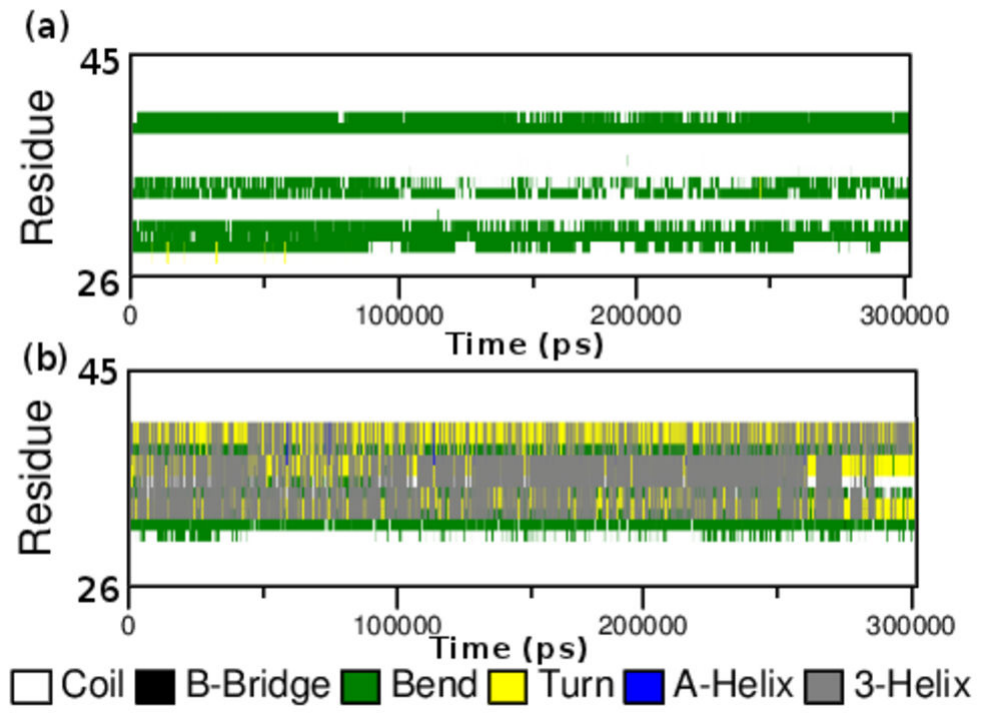
Time evolution of the secondary structural elements of the protein at 300 k (DSSP classification). (a) Native RAC1 Switch I region and (b) Mutant RAC1 Switch I region.

 The presence of higher constrains in the mutant as compared to the native RAC1 structure was further evaluated by the PCA analysis. A better view of dynamic mechanical properties of the investigated system had been obtained by using ED analysis. To check the presence of higher constrains in the mutant as compared to the native RAC1 structure and to support our MD simulation result, the large-scale collective motions of the native and mutant protein using ED analysis. The dynamics of two proteins is best achieved via characterization of its phase-space behaviour. The eigenvectors of the covariance matrix are called its principle components. The changes of particular trajectory along each eigenvector were obtained by this projection. The spectrum of the corresponding eigenvalues indicated the level of fluctuation and dynamic behaviour of protein molecule in the system and was basically confined within the first two eigenvectors. The projection of trajectories obtained at 300 K onto the first two principal components (PC1, PC2) showed the motion of two proteins in phase space and the two features were very apparent from these plots. Firstly, the clusters were well defined in native than mutant. Moreover, the mutant covered a smaller region of phase space particularly along PC1 plane than native and it is depicted in Figure 4. On these projections, we saw clusters of stable states. Our result showed higher range of eigenvector trajectory covered by mutant as compared to the native (Figure 4).

 The RMSF results have indicated that the fashion of conformational deviation induced in the Switch I region of RAC1 is distinct from the overall deviation of the mutant structure as compared to the native. Thus we investigated RMSD and NHbond in the Switch I region. Native and mutant RAC1 Switch I region showed similar type of deviation till ~95 ns (Figure 5). After ~ 95 ns, native and mutant RAC1 protein switch I region showed different deviation pattern up to the end of the simulation. Abrupt rises in the RMSD values were observed after 95 ns in mutant, reaching up to its highest value of 0.4 nm at ~200 ns (Figure 6). At the end of simulation, mutant showed RMSD value of 0.32 nm whereas native showed RMSD value of 0.22 nm. Moreover, a notable loss of NHbond formation was observed in the Switch I region of the mutant structure. The average number of NHbond in native was 36 whereas in mutant it was 24. This significant loss of NHbond clearly explains the cause of rise in flexibility and the overall RMSD score in mutant Switch I region.

 Additional information on the structural flexibility of RAC1 proteins is obtained by the analysis of time-dependent secondary structure fluctuations. Figure 7a,b shows the secondary structural elements as a function of simulation time. Figure 7 reveals that coil and bends are observed in native protein during simulation time period. Compared to native, mutant Switch I region showed significant structural changes between a region of residues 26-45 during simulation. Between residues 26-45, native RAC1 Switch I region showed more coil–coil conformation than mutant. In native structure the coil conformation in the region 30-41 slowly appeared in turn and 3-Helix conformation in the mutant. Between residues of 39-41, bends change to turns conformation in mutant. There were no significant changes observed between residues of 26-28 and 42-45 in native and mutant except a few bend conformation changes to coil conformation for 28^th^ residue. After residues of 30^th^ till 41^st^, in mutant 3-Helix conformations were dominated over coil and bend conformations. Such rapid conformation shifting along with the significant loss in NHbond formation, accompanied in the loss of functional activity of RAC1 protein which in turn induced cancer-associated phenotypic consequences. Furthermore, we investigated if there is any significant loss in the NHbond formation in amino acid residues important for the protein interactions. Our results depicted that the amount of NHbond were greater in most of these amino acids the mutant structure as compared to the native. It clearly indicates that the mutation did not show any damaging consequences on the protein interactions of RAC1, instead it might have improved the interaction affinity with proteins such as RhoGDI. 

## Conclusion

 Point mutations are widely studied in several cancer cases. An in-depth knowledge of the functional and structural impact of these point mutations are required to synthesize an appropriate drug molecule against such emerging cancer cases. Due to the rapidly evolving computational platforms, it has become easier to investigate such consequences at the atomic level. In this work we focused on elucidating the impact of RAC1 protein F28L mutation on the conformational behavior of the protein. Further we investigated the changes in hydrogen bond formation and flexibility of the functional regions. The overall results suggested that the mutation has significantly contributed towards the loss of stability of the RAC1 protein Switch I region, which might be the crucial reason behind the loss of GTPase activity of the protein. Since the loss of stability in the Switch I region is likely to govern the associated cancer cases, targeting it with heavy inhibitor molecules can be a promising approach. It can be further accompanied by the type I pyrrole-indolinone inhibitors targeting the functional regions in their out conformation. Our result presents a valuable insight into the oncogenic transformation of RAC1 protein and it will be very useful in designing therapeutics against such cases. 
